# Effect of thickness and surface finish on light transmission through zirconia-reinforced lithium silicate and lithium disilicate dental ceramics

**DOI:** 10.1590/0103-644020256572

**Published:** 2025-12-08

**Authors:** Stella Sueli Lourenço Braga, Richard Bengt Price, Stefan Mikhail Juckes, Braden Sullivan, Maria Tereza Hordones Ribeiro, Carlos José Soares

**Affiliations:** 1 Department of Operative Dentistry and Dental Materials, Dental School, Federal University of Uberlândia, Uberlândia, Minas Gerais, Brazil; 2 Department of Dental Clinical Sciences, Dalhousie University, Halifax, Nova Scotia, Canada

**Keywords:** ceramics, thickness, surface finish, light transmission, beam profile

## Abstract

This study evaluated the influence of thickness and surface finish on the light transmission of specific wavelengths of light through ceramics. Blocks of a zirconia-reinforced lithium silicate (Celtra Duo, Dentsply Sirona) and lithium disilicate glass-ceramic (IPS e.max CAD, Ivoclar Vivadent) were sectioned into slices (0.25 - 3.30 mm thick) and prepared following their manufacturers’ instructions. The surface gloss and light transmission through the Celtra Duo were measured: first without treatment, then after polishing, after polishing with diamond paste, and finally after glazing. Transmission through e.max was measured only after glazing. The % transmission and light attenuation coefficients were measured through each thickness at: 396, 406, 447, 454, 465, and 476 nm using a spectrometer. The beam profiles were measured and qualitatively analyzed. Data were subjected to 1-way ANOVA and Tukey HSD test (α = 0.05). Only 6% of the light at 396 nm and 21 to 26% at 476 nm passed through 1.5 mm of both ceramics. Almost none of the violet light and only 6% and 9% of the blue light could be detected through 3.0 mm of each ceramic. At the longer wavelengths, more light was transmitted through Celtra Duo. When glazed, Celtra Duo had higher surface gloss compared to e.max. As the ceramic thickness increased, there was a logarithmic decrease in light transmission. The violet light was more attenuated than the blue light. No correlation was found between the surface gloss and the amount of light transmitted, irrespective of the glass ceramic type.

## Resumo

O objetivo deste estudo foi avaliar a influência da espessura e do acabamento superficial na transmissão de luz de diferentes comprimentos de onda através de diferentes cerâmicas. Blocos de dissilicato de lítio reforçado com zircônia (Celtra Duo, Dentsply Sirona) e vitrocerâmica de dissilicato de lítio (IPS e.max CAD Ivoclar Vivadent) foram seccionados em fatias (0,25 - 3,30 mm de espessura) e preparados seguindo as instruções do fabricante. O brilho da superfície e a transmissão de luz através do Celtra Duo foram medidos: sem tratamento, após polimento, após polimento com pasta de diamante e após glazeamento. A transmissão de luz através do e.max foi medida somente após o glazeamento. Os coeficientes de transmissão em % e atenuação de luz foram medidos através de cada espessura nos comprimentos de ondas de: 396, 406, 447, 454, 465 e 476 nm usando espectrômetro. Os perfis de feixe foram medidos e analisados qualitativamente. Os dados foram submetidos ao teste ANOVA de 1 via e ao teste Tukey HSD (α = 0,05). Apenas 6% da luz no comprimento e onda de 396 nm e 21 a 26% comprimento e onda de 476 nm passaram por 1,5 mm de ambas as cerâmicas, e quase nenhuma luz violeta e apenas 6% e 9% da luz azul puderam ser detectadas através de 3,0 mm de cada cerâmica. Nos comprimentos de onda maiores, mais luz foi transmitida através da cerâmica Celtra Duo. Quando aplicado glaze, o Celtra Duo teve maior brilho de superfície em comparação ao IPS e.max CAD. À medida que a espessura da cerâmica aumentou, houve diminuição logarítmica na transmissão de luz. A luz violeta foi mais atenuada que a luz azul. Não foi encontrada correlação entre o brilho da superfície e a quantidade de luz transmitida, independentemente do tipo de cerâmica.

**Figure:**
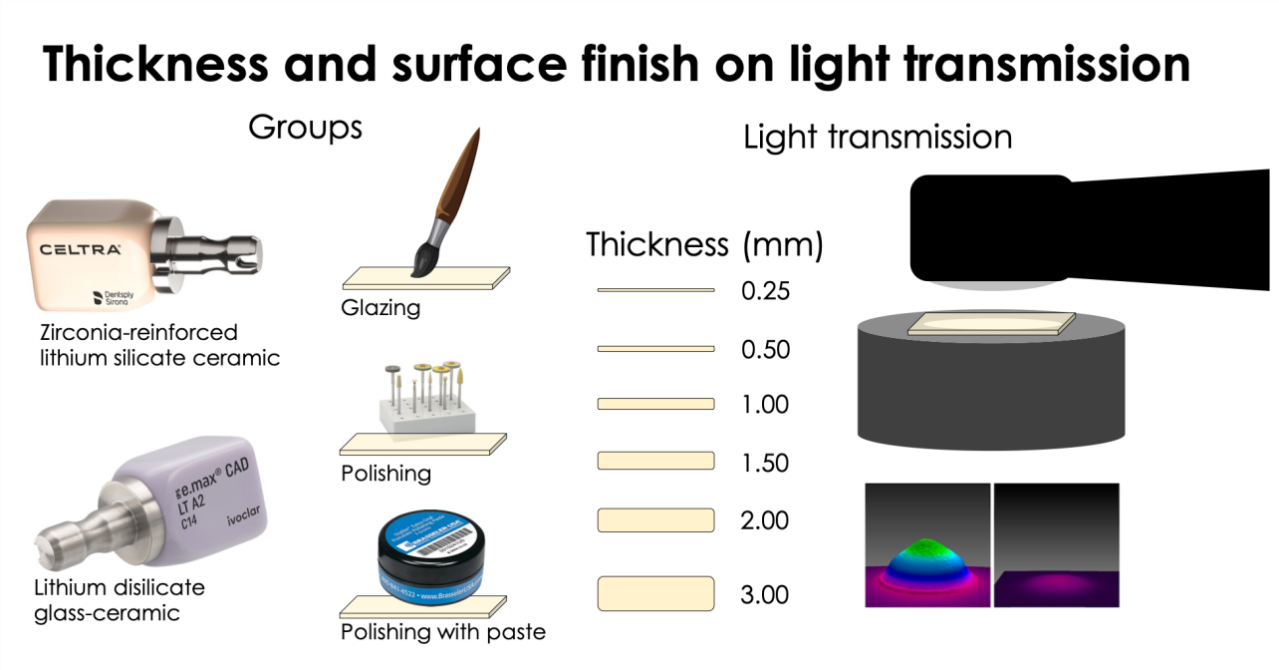

## Introduction

The evolution in Computer-Aided Design and Computer-Aided Manufacturing (CAD/CAM) has stimulated dentists to produce faster indirect restorations. However, the success of CAD/CAM restorations is highly dependent on the luting procedure ^1^ ^,^ ^2^. Lithium disilicate glass-ceramics are often used because they possess excellent optical and mechanical properties; however, this can reduce the amount of light that reaches the resin cement ^3^. A translucent zirconia-reinforced lithium silicate material has been developed, offering a wide range of options and varieties for enhancing esthetics ^4^. As expected, this light attenuation has already been reported to increase with the ceramic thickness ^3^ ^,^ ^5^ ^,^ ^6^ ^,^ ^7^, and a reduction in the amount of light received by the resin may negatively affect the polymerization process of the resin cement ^3^ ^,^ ^5^. It has also been previously reported that the shorter wavelengths of light (violet light) do not penetrate as well as the longer wavelengths (blue light) ^8^ ^,^ ^9^. This lack of penetration through the ceramic may affect the degree of conversion and microhardness of resin cements using photoinitiators that require these shorter wavelengths of light ^3^ ^,^ ^5^. The effect of delivering this additional violet light and energy may be only to cause an unnecessary rise in temperature.

The material of choice for bonding all-ceramic restorations to the tooth preparation is usually a light-cured, heated, regular paste resin composite, resin cement, or a dual-cured resin cement ^1^ ^,^ ^2^ ^,^ ^10^. These resin materials contain one or more photoinitiator systems that are activated by different light wavelengths ^1^ ^,^ ^11^ and they require sufficient energy to achieve adequate resin polymerization ^1^. However, some other initiators, such as diphenyl ^2^ ^,^ ^4^ ^,^ ^6^ trimethylbenzoyl) phosphine oxide (TPO), are sensitive only to shorter wavelengths of light ^12^. Light-emitting diode (LED) - light-curing units (LCUs) that emit multiple-wavelength peaks of light, covering both the violet and blue ranges, have been developed. Unfortunately, little of the violet light from these multiple-wavelength LCUs reaches the resin cement ^5^ ^,^ ^6^, and unless they are well-designed, some LCUs deliver an inhomogeneous light output ^13^. However, the effect of ceramic thickness and surface finish ^14^ on the beam homogeneity after passing through the ceramic, as well as the specific wavelengths of violet and blue light transmitted through different types of CAD/CAM ceramics, requires further investigation. The effects of a polished surface compared to a glazed dental ceramic on light reflection, internal dispersion, and transmission are unknown, which can result in less energy being delivered to the resin cement. Although most CAD/CAM ceramic restorations receive a final glazed surface created by an oven-firing process, some chairside CAD/CAM ceramics, such as Celtra Duo, can just be polished ^15^. These finishing and polishing procedures have been reported to improve the esthetics of the milled ceramic and are expected to result in similar refraction and reflection characteristics to those of a natural tooth ^15^. Additionally, highly polished and smooth surfaces will decrease bacterial adhesion and reduce abrasive wear on the antagonist teeth or restorations ^16^. Consequently, it is recommended that the ceramic be polished to a high gloss after any adjustment to ensure optimal clinical performance.

Thus, this study investigated the effect of thickness and surface finish on the light transmission of two different CAD/CAM ceramic types at six discrete wavelengths within the violet and blue wavelength ranges. The hypotheses were that: Different thicknesses and surface finishes would not influence the light transmission through the two types of ceramic; the light transmission through the ceramics would be the same at the different wavelengths tested, and; The surface treatments would not affect the surface gloss of Celtra Duo.

## Materials and methods

### Study design

Two dental ceramic CAD/CAM materials were used: Celtra Duo, a zirconia-reinforced lithium silicate (Dentsply Sirona, York, PA, USA), and IPS e.max CAD, a lithium disilicate glass-ceramic (Ivoclar Vivadent, Schaan, Liechtenstein). The ceramics and their composition, as provided by their manufacturers, are described in Table 1. Both ceramics were described as ‘low translucency A2’ shade. The surface gloss was measured to determine if the finish affected how light was transmitted through the ceramic. The light transmission through the various ceramic thicknesses was evaluated at 396 nm, 406 nm, 447 nm, 454 nm, 465 nm and 476 nm wavelengths from three contemporary light-emitting diode (LED) LCUs: a single-peak light, SmartLite Focus (Dentsply Sirona), that emitted light peaking at 476 nm, two broad-spectrum multipeak LCUs VALO Grand (Ultradent, South Jordan, UT, USA), that emitted light that had peak wavelengths at 396, 447 and 465 nm and the Bluephase Style (Ivoclar Vivadent) that emitted peak wavelengths of light at 406 and 454 nm. The amount of light transmitted at these different wavelengths through both types and the six different ceramic thicknesses was determined using a laboratory-grade spectrometer (USB-4000, Ocean Insight, Orlando, FL, USA). A beam proﬁler (Ophir-Spiricon, Logan, UT, USA) was used to evaluate the effects of light beam inhomogeneity on the light distribution through the ceramic thicknesses at the different light wavelengths. Five measurements were performed for each of the different ceramic thicknesses.

**Table 1:** Ceramics used in the study (data provided by the manufacturers).

Ceramic Material	Classification	Composition	Lot No.	Manufacturer
IPS e.max CAD LT A2	Lithium disilicate glass-ceramic	SiO_2_ 57-80%, Li_2_O 11-19%, K_2_O 0-13%, P_2_O_5_ 0-11%, ZrO_2_ 0-8%, ZnO 0-8%, Al_2_O_3_ 0-5%, MgO 0-5%, colouring oxides 0-8%	X42573	Ivoclar Vivadent, Schaan, Liechtenstein
Celtra Duo LT A2	Zirconia-reinforced lithium silicate ceramic	SiO_2_, Li_2_O, ZrO_2_ (~10%), P_2_O_5_, Al_2_O_3_, K_2_O, CeO_2_, pigments	18031165	Dentsply Sirona, York, USA

Abbreviations: Al_2_O_3_, aluminum trioxide; CeO_2_, cerium dioxide; K_2_O, potassium oxide; Li_2_O, lithium oxide; MgO, magnesium oxide; P_2_O_5_, phosphorus pentoxide; SiO_2_, silicon dioxide; ZnO, zinc oxide; ZrO_2_, zirconium dioxide.

### Sample preparation

CAD/CAM blocks of both ceramics were sectioned into slices that ranged from 0.25 to 3.30 mm thick using a diamond disc and a precision saw (IsoMet 15LC, Buehler, Lake Bluff, IL, USA). The manufacturer's instructions state that Celtra Duo can be either polished or glazed, while IPS e.max must always be crystallized and glazed before cementation. Thus, the Celtra Duo LT A2 slices were first evaluated without any surface treatment. Then they were randomly allocated to receive the finishing treatments recommended by the manufacturer. Since IPS e.max must always be crystallized in a furnace before it is delivered to the patient, the slices of IPS e.max LT A2 were first evaluated without any surface treatment, only to measure their initial surface gloss. The light transmission was only measured after one side had been glazed, following the manufacturer's instructions.

*Polishing:* The ceramic slices were cleaned using water and dried using an air spray. One side of the slice underwent a pre-polishing step using a diamond polisher (#53 5990 2103, Celtra Set, TwisTec, DeguDent, Dentsply Sirona). The final polishing step used a diamond polisher (#53 5990 2104, Celtra Set, TwisTec, DeguDent, Dentsply Sirona) at 10,000 rpm. All slices were then cleaned with detergent and water to remove the polishing residues.

*Polishing with diamond paste:* The Celtra Duo slices were cleaned, and one side was polished following the instructions as the polishing group. However, the final step involved applying a fine diamond paste (Dialite Extra-Oral Polishing Paste, Brasseler USA, Savannah, GA, USA) to a soft Robinson brush (#11/16 SOFT, Brasseler USA) at a speed of 8,000 rpm, using low pressure to achieve a high-gloss finish.

*Glazing:* One side of the Celtra Duo LT A2 slices was treated following the manufacturer's instructions (Dentsply Sirona). The slices were covered with Universal Stain and glazed, followed by Universal Overglaze (Dentsply Sirona). The ceramic discs were fired twice using the press furnace (Programat EP3000, Ivoclar Vivadent) to glaze the slices.

In accordance with the manufacturer's instructions (Ivoclar Vivadent), the IPS e.max CAD LT A2 slices were only evaluated after glazing. One side of each ceramic slice was treated following the manufacturer’s instructions. The crystallization and glazing were carried out in one step using IPS e.max CAD Crystallization/Glaze paste and stain paste (Ivoclar Vivadent) in a press furnace (Programat EP 5010, Ivoclar Vivadent) for 20 minutes. All finishing and polishing were performed manually by a single, calibrated operator.

### Surface gloss

The surface gloss [GU] was measured with a Novo-Curve glossmeter (Rhopoint Instruments Ltd, Hastings, Sussex, UK) using a 60° angle of illumination and a 4.5 mm aperture. The equipment was calibrated with a standard plate (93.3 GU) provided by the manufacturer before making the measurements. The center of the sample was placed on the aperture and adjusted for each measurement. The ceramic surface gloss was measured four times in different locations, with the disc in two orientations (North-South and East-West). These eight measurements were then averaged to provide a gloss value for each surface.

### Light transmission through the ceramics

**Figure 1:**
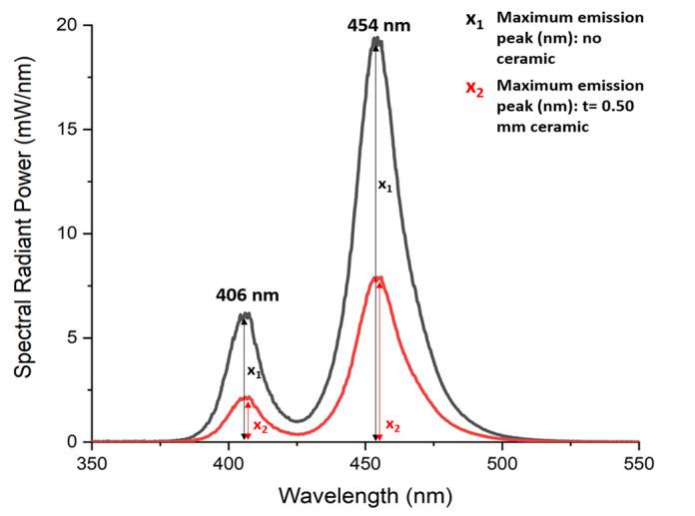
Maximum peak wavelengths at 396, 406, 447, 454, 465, and 476 nm were used to calculate the light transmission through ceramics. The transmission of light was calculated by dividing the power passing through the ceramic (x_2_) by the maximum power recorded at that wavelength in the absence of ceramic (x_1_).

The total light transmission through both ceramics, of different thicknesses and surface finishes, from each LCU was measured through a 12 mm aperture into a six-inch integrating sphere (LabSphere, North Sutton, NH, USA), connected to a fiber-optic spectrometer (USB 4000, Ocean Insight). The tip of the LCU was positioned 0 mm away from and parallel to the specimen so that the light incidence was perpendicular to the specimen. All measurements were made similarly, with the light tip positioned at 0 mm, so that the effects of light reflection could be minimized. For each condition, three measurements of the spectral radiant power (mW/cm²/nm) transmitted through the specimens were made between 350 and 550 nm. The percentage of light transmission was evaluated by measuring changes in the heights of each discrete peak wavelength at 396, 406, 447, 454, 465, and 476 nm. The transmission was calculated by dividing the power measured through the ceramic (x_2_) by the maximum power recorded at that wavelength in the absence of ceramic (x_1_) (Figure 1).

The amount of violet and blue light was fitted using Microsoft Excel software (Microsoft Corporation, Redmond, Washington, USA), with the following formula according to the Beer-Lambert law:

$$T\left(t\right)=T_{O}e^{\frac{-t}{d_{o}}}$$

Where T is transmission as a function of distance, T_o_ is the transmission at zero thickness, t is the thickness, and d_o_ is a characteristic decay length called the extinction length.

Using the discrete peak wavelengths at 396, 406, 447, 454, 465, and 476 nm, the energy (J) delivered at each peak was calculated by multiplying the power (W) at these peaks by an exposure time of 10 seconds. The radiant exposure (J/cm²) delivered at each wavelength peak through the different thicknesses of glazed ceramics was then calculated by dividing these energy values by the effective tip area (cm²) of each LCU.

### Beam profile of the light transmitted through the ceramics

The light beam profiles of the light transmitted through representative 0.25 mm and 1.50 mm thick ceramic slices were measured using a laser beam profiler camera (USB-L070, Ophir-Spiricon). The LCU guide tip was placed against the polished or glazed side of the ceramic slice, and the transmitted light beam was examined from the other unfinished side using a digital camera with a 50 mm focal length lens. A blue filter (International Light Technologies, Peabody, MA, USA) was used to flatten the spectral response of the CCD camera. Images of the transmitted light beam were taken through narrow bandpass filters that had a 10 nm full-width half maximum bandwidth centered at either 400 nm (violet) or 460 nm (blue) (#65-132 and #88-010, Edmund Industrial Optics, Barrington, NJ, USA) so that only the violet or blue light images were recorded using BeamGage Professional 6.14 software (Ophir-Spiricon).

### Light Attenuation Coefficient

To evaluate how the transmitted light from each LCU decreased as it passed through the specimens, the attenuation coefficient (AC, mm^−1^) was estimated using the measured light at different thicknesses based on the Beer-Lambert law:

$$I\left(z\right)=Io\;e^{-az}$$

Where Io is the initial light intensity (the measure of light in the absence of the specimen), α is the attenuation coefficient, and z is the specimen thickness. Since all specimens were examined under the same conditions, the light reflection effects were considered to be uniform across all specimens.

### Statistical analysis

The light transmission through Celtra Duo LT A2 was analyzed using a two-way analysis of variance (ANOVA) followed by a post-hoc Fisher’s exact test to compare the effects of thickness and the different surface treatments at each wavelength. The light transmission through IPS e.max CAD LT A2 was determined using a one-way ANOVA followed by a post-hoc Fisher's exact test to compare the effects of the ceramic thickness at each wavelength. A one-way ANOVA was used to analyze the surface gloss data for Celtra Duo LT A2. The surface gloss data for both ceramics, first without any treatment and then after glazing, were analyzed using a two-way ANOVA. Multiple comparisons were made using Tukey’s test. For each ceramic, the Pearson correlation test was used to determine if there was any correlation (R^2^) between the surface gloss and the light transmission at 396, 406, 447, 454, 465, and 476 nm. All tests used an α = 0.05 significance level, as determined by SigmaPlot version 13.1 (Systat Software, Inc., San Jose, CA, USA). The beam profiling images were analyzed qualitatively.

## Results

### Surface gloss

**Figure 2:**
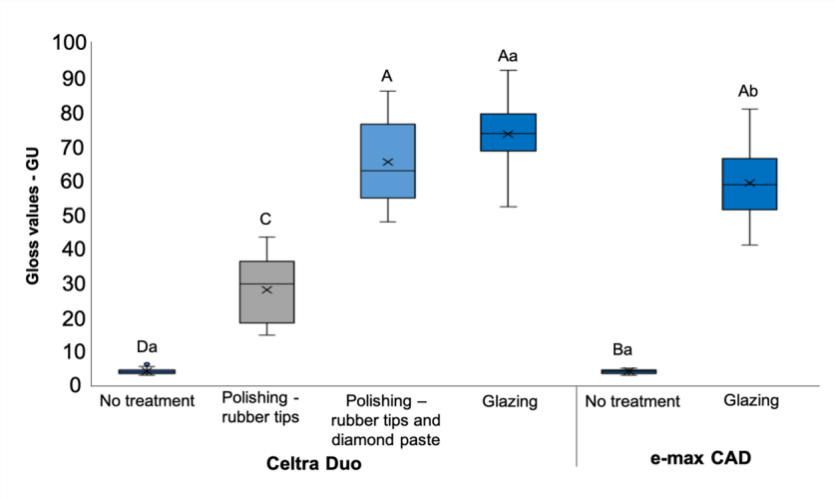
Surface gloss values for both ceramics after different finishing treatments. Upper case letters are used for comparing different finishing treatments for each ceramic; lower case letters are used for comparing the same finishing treatments on the different ceramics (p<0.05).

The ceramic slices were 0.25, 0.5, 1.0, 1.5, 2.0, and 3.0 mm thick. There was a significant interaction between the finishing procedures and the type of ceramic (p<0.001). The Celtra Duo LT A2 glazed ceramic had higher surface gloss values than the IPS e.max CAD LT A2 after glazing. There was no significant difference in gloss between the samples polished with diamond paste and those glazed for Celtra Duo LT A2 (Figure 2). A weak correlation was found between gloss values and the percentage of light transmission through ceramic, regardless of the evaluated wavelength (R² < 0.25, p < 0.001) (Table 2).

**Table 2:** Pearson correlation between surface gloss value and the percentage of light transmission at the six discrete wavelength peaks

Wavelength (nm)	R	R^2^	***P* value**
396	-0.109	0.012	0.223
406	- 0.119	0.014	0.186
447	- 0.147	0.019	0.101
454	- 0.139	0.021	0.119
465	- 0.147	0.025	0.101
476	- 0.149	0.022	0.096

### Light is transmitted through the ceramics

**Figure 3:**
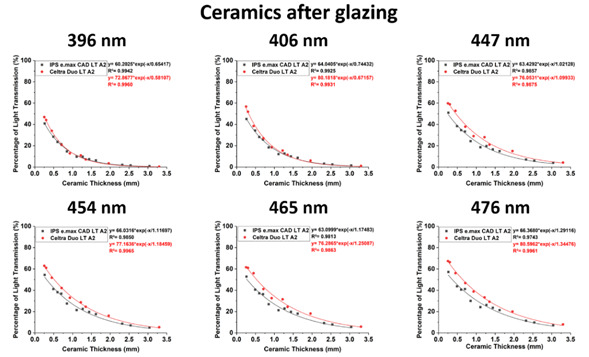
Percentage of light transmission (%) at each wavelength. Note the effect of thickness and wavelength on the transmission of light.

**Figure 4:**
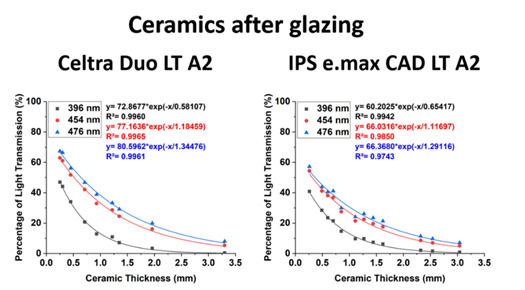
Comparison of the light transmission (%) at representative wavelengths of 396, 454, and 476 nm. Note the effect of thickness and the wavelength on the transmission of light.

The thicknesses of the ceramics and the wavelengths of light had a significant effect on light transmission (p<0.001). There was an exponential decline (R²>0.97, p<0.001) in the amount of light transmitted as the ceramic thickness increased. The lower wavelengths of light were more affected (Figures 3 and 4). However, the various surface finishes did not significantly affect the light transmission at each wavelength. Using a logarithmic curve fit, the predicted amount of violet light transmission at 396 nm through 0.25 mm of IPS e.max CAD LT and Celtra Duo LT A2 was 41% and 47%, respectively. Only 6% of the light at 396 nm passed through 1.5 mm of both ceramics, and almost none (~0%) of the original light at 396 nm could be detected through 3.0 mm of each ceramic (Table 3). At the longer wavelengths of light, more light was transmitted through Celtra Duo LT A2 than through IPS e.max CAD LT (Figure 3). At 476 nm, the percentage of the incident light transmitted through 0.25 mm of IPS e.max CAD LT and Celtra Duo LT A2 was 55% and 67%, respectively. This fell to 21% and 26% through 1.5 mm of ceramic, but only 6% and 9% of the light passed through 3.0 mm of each ceramic, respectively.

A higher radiant exposure was delivered through Celtra Duo LT A2 than through IPS e.max CAD LT at the longer wavelengths (Figure 3). Table 4 shows that at 476 nm, the radiant exposure delivered through 0.25 mm of IPS e.max CAD LT and Celtra Duo LT A2 was 0.051 and 0.059 J/cm², respectively. Through 1.5 mm of ceramic, the radiant exposure was 0.021 and 0.026 J/cm². Notably, only 0.006 and 0.007 J/cm² were delivered through 3.0 mm of each ceramic, respectively. At 396 nm, the calculated radiant exposure (J/cm²) delivered in 10 s through 0.25 mm of IPS e.max CAD LT and Celtra Duo LT A2 was 0.010 and 0.011 J/cm², respectively, and 0.002 J/cm² through 1.5 mm of both ceramics. It was undetectable (0.0) at 396 nm.

**Table 3:** Predicted light transmission (%) at representative 396, 454, and 476 nm wavelength peaks (0 mm = 100%).

Ceramic	Predicted Light Transmission (%)																				
	396							454							476						
	0.25 mm	0.50 mm	1.00 mm	1.50 mm	2.00 mm	3.00 mm	R²	0.25 mm	0.50 mm	1.00 mm	1.50 mm	2.00 mm	3.00 mm	R²	0.25 mm	0.50 mm	1.00 mm	1.50 mm	2.00 mm	3.00 mm	R²
IPS e.max CAD LT A2	41	28	13	6	3	1	0.994	53	42	27	17	11	5	0.985	55	45	31	21	14	6	0.974
Celtra Duo LT A2	47	31	13	6	2	0	0.996	62	51	33	22	14	6	0.996	67	56	38	26	18	9	0.996

Abbreviations: R² is the coefficient of determination which is the proportion of the variance in the dependent variable that is predictable from the independent variable; 396 nm is the peak of the short wavelength of light emitted from the VALO Grand; 454 nm is the peak of the long wavelength of light emitted from the Bluephase Style; 476 nm is the single-peak blue light emitted from the SmartLite Focus.

**Table 4:** Radiant exposure (J/cm²) that would be delivered in 10 s through glazed ceramic thicknesses at representative 396, 454, and 476 nm peak wavelengths.

Ceramic	Radiant exposure delivered in 10 s (J/cm²)																	
	396						454						476					
	0.25 mm	0.50 mm	1.00 mm	1.50 mm	2.00 mm	3.00 mm	0.25 mm	0.50 mm	1.00 mm	1.50 mm	2.00 mm	3.00 mm	0.25 mm	0.50 mm	1.00 mm	1.50 mm	2.00 mm	3.00 mm
IPS e.max CAD LT A2	0.010	0.007	0.002	0.002	0.000	0.000	0.035	0.027	0.014	0.013	0.006	0.003	0.051	0.039	0.022	0.021	0.010	0.006
Celtra Duo LT A2	0.011	0.008	0.003	0.002	0.001	0.000	0.041	0.034	0.022	0.016	0.011	0.003	0.059	0.049	0.034	0.026	0.018	0.007

Abbreviations: 396 nm is the peak of the short wavelength emitted from the VALO Grand; 454 nm is the peak of the long wavelength emitted from the Bluephase Style; 476 nm is the single-peak blue emitted from the SmartLite Focus

### Beam profile of the light transmitted through the ceramics

**Figure 5:**
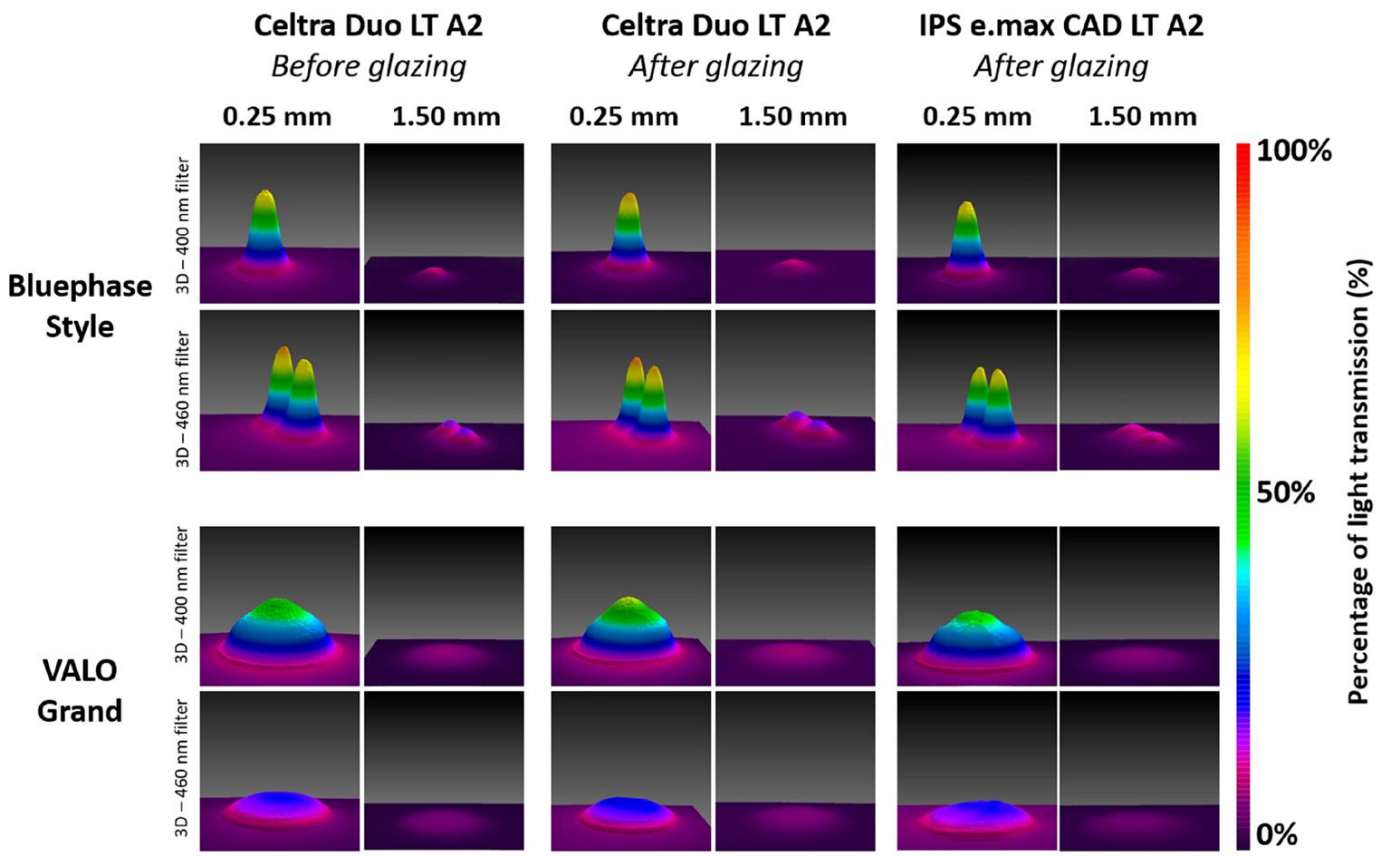
Three-dimensional representations of the beam profile recorded through a 400 nm bandpass filter (that allowed only violet light to pass), and through a 460 nm bandpass filter (allowing only blue light to pass) from the standard output mode of Bluephase Style, and VALO Grand through Celtra Duo LT A2 (before and after glazing), and through IPS e.max CAD LT A2 (after glazing). The images taken through the 400 nm and the 460 nm bandpass filters are on their percentage scales at each wavelength range.

The color-coded representations of the beam profile recorded from the Bluephase Style and VALO Grand through Celtra Duo LT A2 (before and after glazing) and IPS e.max CAD LT A2 (after glazing) through the narrow bandpass filters are shown in Figure 5. The images taken through the filters are all on the same scale relative to the maximum light transmission from the Bluephase Style at that wavelength range (400 or 460 nm).

The beam profile images showed that the light beam inhomogeneity from the LCU was very evident through 0.25 mm of both ceramics and remained present through 1.5 mm. No light was detected through 3.0 mm of both ceramics. The Bluephase Style had a heterogeneous beam profile, with two spots of light representing the three LEDs in this LCU (two blue LEDs and one violet LED). The VALO Grand had a more homogeneous beam profile, demonstrating no interference from the position of the 4 LEDs in this LCU (3 blue - 2 465 nm and 1 445 nm LEDs, and one violet 405 nm LED) on light homogeneity. The images support the light transmission measurements and demonstrate the effects of beam inhomogeneity on light transmission, the influence of ceramic thickness on the amount of transmitted light, and how shorter wavelengths are more affected than longer wavelengths of light. The beam profile images of the Celtra Duo confirmed higher blue light transmission compared to IPS e.max CAD LT (Figure 5).

### Light Attenuation Coefficient

Less light was transmitted through IPS e.max CAD LT A2 after glazing compared to Celtra Duo LT A2 after it had been glazed. The attenuation coefficients of the emitted light for all wavelengths with the interposition of a slice of glass-ceramic material are reported in Table 5. The attenuation coefficient was greater for the lower wavelengths (396 and 406 nm), regardless of the glass ceramic type and surface treatment (p < 0.001). IPS e.max CAD had a significantly lower attenuation coefficient than Celtra Duo LT at the 396 nm wavelength.

**Table 5:** Calculated attenuation coefficient at the 396, 406, 447, 454, 465, and 476 nm peak wavelengths.

Glass-ceramic	Surface finish	Peak Wavelength (nm)					
		396	406	447	454	465	476
Celtra Duo LT A2	Without any treatment	1.48 ^Cb^	1.18 ^Ba^	0.82 ^Aa^	0.75 ^Aa^	0.74 ^Aa^	0.68 ^Aa^
	After polishing	1.52 ^Cb^	1.24 ^Ba^	0.84 ^Aa^	0.77 ^Aa^	0.75 ^Aa^	0.69 ^Aa^
	After polishing with diamond paste	1.51 ^Cb^	1.26 ^Ba^	0.83 ^Aa^	0.76 ^Aa^	0.74 ^Aa^	0.68 ^Aa^
	After glazing	1.59 ^Cb^	1.27 ^Ba^	0.86 ^Aa^	0.80 ^Aa^	0.77 ^Aa^	0.70 ^Aa^
IPS e.max CAD LT A2	After glazing	1.37 ^Ca^	1.24 ^Ba^	0.90 ^Aa^	0.84 ^Aa^	0.79 ^Aa^	0.73 ^Aa^

Different letters express significant differences. Capital letters are used to compare different wavelengths (nm); lower-case letters are used to compare surface finishes.

## Discussion

As the thickness of the ceramic slices increased, there was a rapid logarithmic decay in the amount of light transmitted through the different types of ceramic. Notably, the finishing treatments did not impact the light transmission. Therefore, the first hypothesis was rejected.

At all the wavelengths of light that were evaluated as the ceramic thickness increased from 0.25 to 3.0 mm ^12^ ^,^ ^17^, the light was logarithmically attenuated (R²>0.97). The amount of attenuation was greater at the shorter wavelengths of light due to their lower transmittance as the thickness increased, because these lower wavelengths of light are scattered more through the ceramic ^12^. Thus, the second hypothesis was rejected. These aspects may negatively affect the light activation of resin cements that use Norrish Type I photoinitiators, as they require shorter wavelengths of light ^12^.

Although the blue light emitted from different LCUs may appear the same to the human eye, when measured using a spectrophotometer, most LCUs emit very different emission spectra ^1^. The present study evaluated the radiant exposure at six specific peak wavelengths, namely 396, 406, 447, 454, 465, and 476 nm (Figure 3 and Table 4) that could be identified from the three different LCUs. These wavelengths encompass the wide range of wavelengths emitted by contemporary LED-based LCUs.

The Celtra Duo has 10% zirconia incorporated into the lithium silicate (LiSi_2_) glass matrix, resulting in smaller silicate crystals that lead to higher translucency and glass content compared to conventional lithium disilicate glass-ceramics ^18^. Celtra Duo showed higher translucency than IPS e.max CAD, but only when polished ^18^. Less light was transmitted, in the beam profile images of the glazed Celtra Duo. The attenuation of IPS e.max was lower than that of Celtra Duo at the lower wavelengths (396 nm) which may have been due to the zirconia present in Celtra Duo. This study confirmed the results from previous studies that evaluated only the broader violet and the blue regions of the emission spectrum ^19^, or the full range of wavelengths from the LCU ^20^. These studies also reported that different ceramic thicknesses affected the radiant exposure received by the resin cement. However, because the present study examined six discrete wavelength ranges, it was able to demonstrate that as the wavelength decreases, very little light is transmitted through the ceramic.

Light-cured resin cements have already been reported to achieve adequate polymerization only when the ceramic is at most 1.0 mm thick ^1^, and the mechanical properties of light-cured and dual-cured resin cements have been reported to be unaffected when the ceramic is between 0.3 and 0.9 mm thick ^1^. Other studies have reported that the ceramic should be no more than 3.0 mm thick for both light-cured and dual-cured resin cements ^21^. In the present study, the percentage of light transmitted showed that only 31% and 38% of the light was transmitted through 1 mm thick IPS e.max CAD LT A2 and Celtra Duo LT A2, respectively, at the longer wavelengths, and only 13% of the light was transmitted through both of these ceramics at the shorter wavelengths. The beam profile images (Figure 5) confirmed that very little light was transmitted through the 1.5 mm-thick glazed ceramics. Many clinicians heat regular viscosity light-cured resin composite to cement the ceramic restoration without considering the thickness of the restoration and the fact that sufficient light energy must reach the resin composite below the ceramic. Therefore, the present study supports the information reported on previous studies that dual-curing resin cements should always be used when the ceramic thickness is 1.5 mm or more ^2^ ^,^ ^22^. Figure 5 also shows that the effects of light beam inhomogeneity are very evident through 0.25 mm of ceramic and can still be seen through 1.5 mm of both ceramics.

The method used in this study to evaluate the logarithmic decay of transmission with ceramic thickness is based on a formula according to the Beer-Lambert law. The effect of reflection from the surface of the ceramic discs at each thickness was assumed to be the same for all specimens. From the values reported in Table 3, the clinician or researcher can estimate how much light will be transmitted based on the wavelength of light and the type and thickness of the ceramic, ensuring at least 6 J/cm² is delivered to achieve adequate polymerization of a light-cured resin cement ^20^.

According to the American Dental Association, a surface gloss of 40 to 60 GU is considered adequate for dental restorations, and the polishing technique can alter the surface of indirect restorations ^23^. This gloss level was achieved and exceeded for glazed ceramics and the polished Celtra Duo LT A2 when polished with the diamond paste. This supports a previous study showing that the polished surface of ceramic can be smoother than the glazed ceramic surface ^17^. This level of surface gloss did not affect the light transmission, and only a low correlation was found between surface gloss and the percentage of light transmission (R² < 0.25). Thus, the third hypothesis, that the surface treatments would not affect the surface gloss of Celtra Duo, was accepted. However, the surface treatment did not affect the light transmission for Celtra Duo. During the crystallization of glazed Celtra Duo, this process can negatively impact the bond strength of the resin cement to ceramic when compared with a partially crystallized non-glazed ceramic ^24^.

*In vitro* studies have limitations; in this study, we tested only two specific ceramic materials. Ceramics produced by different manufacturers can exhibit varying opacities, resulting in different light transmissions. Further studies are necessary to determine the effect of light transmission on the polymerization of light-cured resin cements, heated and flowable resin composites, and dual-curing resin cements. However, such studies must also consider the effects of light beam inhomogeneity, particularly where the polymerization of the resin is measured under the light tip (Figure 5), as this inhomogeneity may affect the results depending on the measurement location. Additionally, the present study only utilized low translucency A2 shade ceramics. Therefore, the light transmitted through other types of ceramics with greater opacity, such as 3Y-zirconia and alumina-based ceramics, should be investigated, as the thickness of these ceramics may have a more significant impact on light transmission. Another important consideration is that the present study utilized three high-quality LCUs from major manufacturers. The light attenuation may be greater for LCUs that deliver a lower and more inhomogeneous light to the restoration. However, the clinician should recognize that violet light will always be attenuated more than blue light, and the value of including photoinitiators in the resin cement that require violet light must now be questioned. Clinicians must also recognize that even small increases in the thickness of the ceramic will have a significant effect on the amount of transmitted light. Very little light, especially violet light, is transmitted through more than 1.5 mm of ceramic. Thus, as the thickness of the ceramic increases, the benefit of using multiple peak or polywave curing lights that deliver additional violet light becomes questionable.

## Conclusion

Within the limitations of this *in vitro* study, the following conclusions can be drawn: There is a rapid logarithmic decay in the amount of light transmitted through the ceramic as the thickness increases. This decay occurs much faster for the shorter wavelengths of light. There was no correlation between the surface gloss and the amount of light transmitted, irrespective of the ceramic tested. Celtra Duo-glazed ceramic had higher surface gloss values than e-max CAD ceramic. There was no difference in the surface gloss values between Celtra Duo when polished with diamond paste or glazed.
